# Evaluation of a school-based intervention to promote mental health of refugee youth in Sweden (The RefugeesWellSchool Trial): study protocol for a cluster randomized controlled trial

**DOI:** 10.1186/s13063-020-04995-8

**Published:** 2021-01-28

**Authors:** Natalie Durbeej, Serena McDiarmid, Anna Sarkadi, Inna Feldman, Raija-Leena Punamäki, Reeta Kankaanpää, Arnfinn Andersen, Per Kristian Hilden, An Verelst, Ilse Derluyn, Fatumo Osman

**Affiliations:** 1 Child Health and Parenting (CHAP), Department of Public Health and Caring Sciences, Uppsala University, BMC, Husargatan 3, 753 27 Uppsala, Sweden; 2 Faculty of Social Sciences, Psychology, FI- 30014, University of Tampere, Tampere, Finland; 3 Norwegian Centre for Violence and Traumatic Stress Studies, NO-0409 Oslo, Norway; 4 Faculty of Psychology and Educational Sciences, Department of Social Work and Social Pedagogy, Centre for the Social Study of Migration and Refugees, Ghent University, Ghent, Belgium; 5 School of Education, Health and Social Studies, Dalarna University, 791 88 Falun, Sweden

**Keywords:** Teaching recovery techniques, In-service teacher training, Post-traumatic stress disorder, Refugee youths, School setting

## Abstract

**Background:**

Sweden is home to a large and growing population of refugee youths who may be at risk of mental health problems such as post-traumatic stress disorder (PTSD). Thus, there is a need for interventions that address mental health problems in these populations. Schools have been identified as an ideal setting for delivering such interventions as they offer a non-stigmatizing space and are often central to young refugees’ social networks. The RefugeesWellSchool trial in Sweden will investigate an intervention comprising two programmes: Teaching Recovery Techniques (TRT) and In-service Teacher Training (INSETT), delivered in a school setting, among refugee youth. TRT is a group-based programme for children and adolescents, informed by Trauma-Focused Cognitive Behavioral Therapy (TF-CBT). INSETT is a multi-module course for teachers providing information on trauma and the refugee experience to build teachers’ cultural competence and capacity for supporting refugee youths in schools.

**Methods:**

This trial employs a cluster randomized-control design with two arms: (1) the intervention arm in which the TRT and INSETT programmes are offered (*n* = 350), (2) the wait-list control arm (*n* = 350) in which services are provided as usual until the TRT and INSETT programmes are offered approximately six months later. Data will be collected prior to the intervention, immediately following the intervention, and at three months post-intervention. Outcomes for the trial arms will be compared using linear mixed models or ANCOVA repeated measures as well as the Reliable Change Index (RCI).

**Discussion:**

This study will provide knowledge about the effectiveness of an intervention comprising two programmes: a group-based programme for youth reporting symptoms of PTSD and a training course for teachers, in order to build their competence and ability to support refugee youths in schools.

**Trial registration:**

ISRCTN, ISRCTN48178969, Retrospectively registered 20/12/2019.

**Supplementary Information:**

The online version contains supplementary material available at 10.1186/s13063-020-04995-8.

## Introduction

In 2015, Europe experienced the “refugee crisis” when millions of people were displaced. Displacement has continued and the number of refugees worldwide continues to grow [1]. Thousands of refugees have entered Sweden, including many children and youth [2]. Sweden is generally viewed as successful in its acceptance and reception of refugees, but it still experienced many challenges in providing quality services to refugees, especially during the height of the refugee crisis [3]. Young refugees are an especially vulnerable population. Persistent stress, like that experienced by young refugees facing displacement, threats to their safety, unstable environmental and social conditions, as well as acculturation stress, contribute to the development of mental health problems [4–6]. Additionally, traumatic experiences, such as loss of relatives, experience of persecution and exposure to torture, physical and/or sexual violence, are also highly related to the development of mental health problems [7–9]. A high prevalence of mental health problems including post-traumatic stress disorder (PTSD), depression, and anxiety has been identified in young refugees [7, 10–12]. Thus there is a clear need for programs and interventions that quickly and efficiently address the mental health needs of young refugees.

Interventions have been developed to support mental health, recovery and resilience in refugee populations and schools have been identified as an ideal setting to implement such interventions [13–15]. Functionally, school-based interventions provide a unique opportunity to easily reach youth who may otherwise be isolated or unable to access services [14, 16–19].

Socially, schools are non-stigmatizing spaces, central to a refugee’s social network and a main contact point with the host society. Mental health interventions taking place in a school context may further promote the development of social support networks and positive intercultural relationships while countering stigma and discrimination [17, 18, 20].

Previous studies generally recommend Trauma-Focused Cognitive Behavioral Therapy (TF-CBT) for reducing symptoms of PTSD and other mental health problems in refugee children [21, 22]. An intervention based on TF-CBT is the Teaching Recovery Techniques (TRT) programme, developed by the Children and War Foundation in Norway and the UK [23]. The TRT is a manualised group intervention, including both youth and caregiver sessions, aiming to promote coping and recovery from symptoms of PTSD in children aged eight and above with traumatic experiences. The TRT programme has been used effectively in several contexts such as Palestine and Gaza [24, 25] and post-tsunami Thailand [26]. It has also been used in a Swedish context with 208 unaccompanied refugee minors [27]. This pilot study showed that 84% of the youths reported moderate to severe depression at baseline, and that both depression symptoms and PTSD symptoms were significantly lowered after the intervention. A meta-analysis of 19 studies found that cognitive behavioral therapy interventions delivered in schools are effective in reducing PTSD symptoms among young people, including refugee youth [28]. According to our knowledge, the TRT has not been evaluated in a Swedish school setting.

In addition to TF-CBT interventions targeting youth, teacher interventions might play an important role in promoting refugee youth’s mental health [20, 29–32]. An intervention targeting teachers working with refugee youth is the In-service Teacher Training (INSETT) programme [32, 33], aiming to enhance teachers’ insights into how refugee experiences may affect young people’s psychosocial well-being and school functioning upon resettlement in a new country [29, 32]. It allows teachers to better understand and support young refugees at school through encouraging positive interethnic relationships and strengthening school belonging, as well as fostering supportive interrelationships with parents, caregivers or guardians to promote school involvement. In other words, INSETT seeks to make teachers and schools (more) ‘refugee competent’ [20]. No previous research has evaluated the INSETT programme in a school setting.

To conclude, schools play an important role to promote refugee and migrant youth’s mental health. However, there is limited research on the impact of school-based interventions addressing mental health in refugee youth, especially in the Swedish context [34].

The RefugeesWellSchool trial in Sweden aims to strengthen the evidence base for two school-based programmes: TRT and INSETT, among refugee youth. The objectives of the trial are:

1. To investigate whether the TRT programme, delivered in a school setting, has a positive effect on mental health among youth who screened positive for PTSD symptoms in comparison to services as usual
2. To investigate whether the INSETT programme, delivered in a school setting, has a positive effect on teacher-parent collaboration and teacher multicultural competence in comparison to teachers who receive service as usual
3. To investigate whether the INSETT programme, delivered in a school setting, has a positive effect on mental health, social support and school belonging among youth whose teachers have received the INSETT programme, in comparison to youth whose teachers have not received the programme
4. To investigate whether the caregiver sessions of the TRT programme have a positive effect on parental/guardian mental health in comparison to parents/guardians who have not attended the caregiver sessions of the TRT programme
5. To describe the implementation of the TRT and INSETT programmes using process evaluation
6. To estimate the cost-effectiveness of the two programmes.

It is hypothesized that:

- Youth who screened positive for PTSD symptoms and receive TRT will report fewer mental health problems, especially PTSD symptoms, as well as depression and anxiety symptoms, in comparison to youth who have not received the intervention (the waitlist-control).
- Teachers who receive INSETT will report increased positive teacher-parent relationships and multicultural competence, in comparison to teachers who have not received the intervention (the wait-list control).
- Youth whose teachers received the INSETT will report fewer mental health problems and higher levels of social support and school belonging, in comparison to youth whose teachers have not received the INSETT programme (the wait-list control).
- Parents/guardians who attend TRT as caregivers will report fewer mental health problems, in comparison to parents/guardians who have not received the intervention (the wait-list control).
- Both programmes generate health gains for reasonable costs

## Methods/design

### Study design

**Fig. 1:**
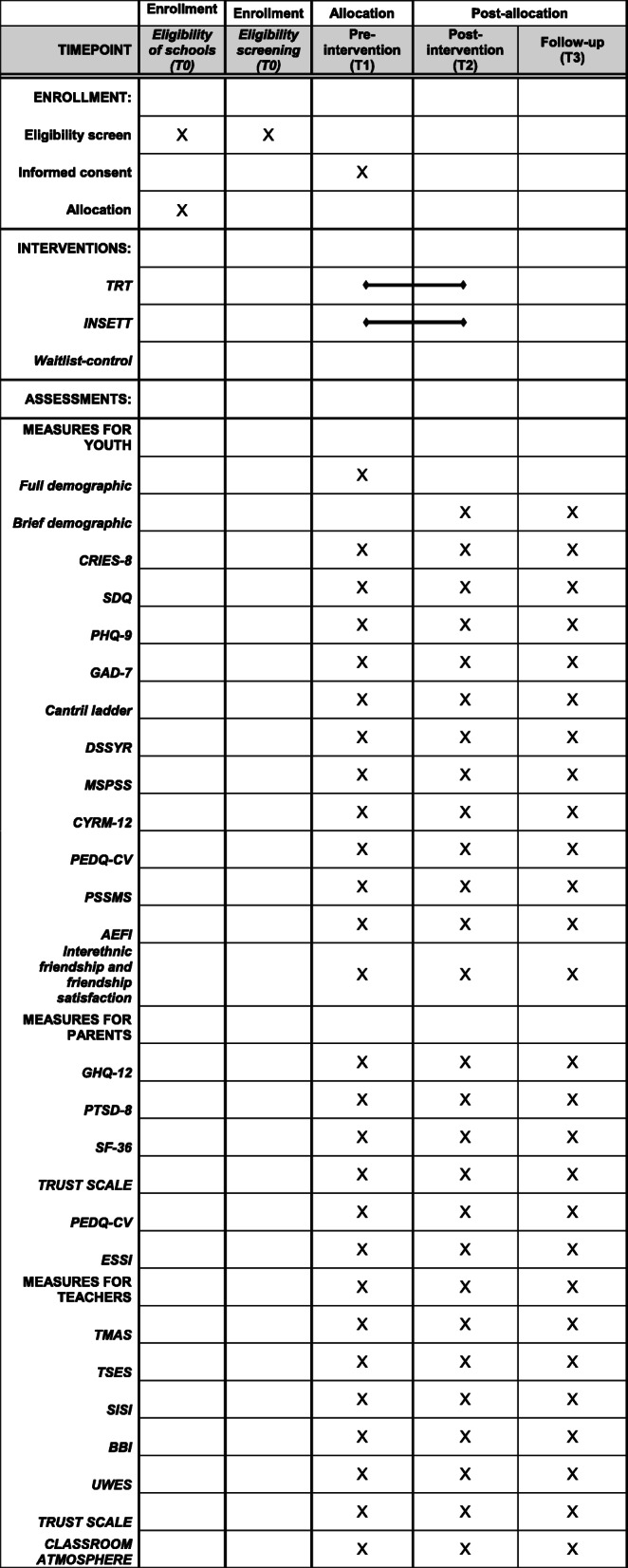
Schedule for enrollment, interventions and assessments

The trial is a cluster randomized-control design using two arms: the intervention arm, which receives the TRT and INSETT programmes, and the wait-list control arm, which receives services as usual until approximately six months after the study begins. The cluster is assigned by school and in an allocation ratio of 1:1. Assessment will take place at three time points: 1) at baseline, prior to the intervention beginning (T1); 2) immediately following the intervention (T2); and 3) three months post-intervention (T3). The trial will run from 2019 to 2021/2022. See Fig. 1 for an overview of assessments. The Standard Protocol Items: Recommendations for Interventional Trials (SPIRIT) checklist is provided as an Additional file. The trial has been approved by the Regional Ethical Review Board in Uppsala (Dnr: 2019–031160), Sweden and registered with an international trial registry (ISRCTN 48178969).

### Setting

The intervention will be implemented in schools that run classes for grades 7–9 and/or introduction classes for newcomer youths. Schools that are multi-ethnic, with the definition that at least 30% of their registered students have a background other than Swedish, will be included. Such schools will be identified using publicly available information on municipal websites. Schools from across Sweden, including rural, suburban, and urban areas, will be contacted.

### Schools and participants

Schools are eligible to participate in the study if they meet the following inclusion criteria:

1. The school consents to participation.
2. The school is multi-ethnic, with 30% of its registered students having a non-Swedish background.
3. The school is a secondary or upper secondary school with students in grades 7–9 and/or in introduction classes for newcomers.

Teachers are eligible to participate if they meet the following criteria:

1. Teacher consents to participation

All youth within participating schools are eligible to participate in the data collection of this study if they meet the following criteria:

1. Youth’s legal guardian consents to participation if he/she is < 15 years.
2. Youth consents to participate if he/she is ≥15 years.

The TRT programme will only be offered to youth who meet the following inclusion criteria:

1. Youth’s legal guardian consents to participation if the youth is < 15 years.
2. Youth consents to participate if he/she is ≥15 years.
3. Youth has been in Sweden less than or equal to 6 years.
4. Youth has screened positive for PTSD symptoms (≥17 points) on the Children’s Revised Impact of Event Scale-8 (CRIES-8) [35].
5. Youth has no other ongoing mental health therapeutic intervention.

Parents/guardians are eligible to participate if they meet the following criteria:

1. Parents/guardians consent to participation.
2. Parents/guardians has a child who participates in the TRT programme.

### Recruitment process

Eligible schools are recruited through phone and email contact with municipalities, school directors, and principals. Schools are provided with information about the study, including study aims and methodology in order to inform their decision to participate. Eligible schools will be allocated to an intervention group or a wait-list control group.

Information meetings will be held for teachers, parents/guardians and youth to recruit participants. All participants will be provided with written information about the study and give their informed consent.

Baseline (T1) assessment with youths will be conducted through an online survey targeting whole classes and T1 assessment with teachers and parents/guardians will be conducted where participants are offered to either fill in the online survey or a paper version of the survey.

All youth will be screened with the CRIES-8 [35] included in the baseline survey, for assessment of PTSD symptoms. The CRIES-8 includes four intrusion and four avoidance items pertaining to a specific traumatic event. Participants report the frequency of the symptoms in the previous week on a scale from 0 (*not at all*) to 1, 3 and 5 (*often*). The CRIES-8 has been found to have strong psychometric properties among children living in contexts where traumatic events are prevalent, including adequate discriminant and convergent validity and test-retest reliability [36]. In addition, it has been shown to have good internal consistency its factor structure confirmed in a sample of unaccompanied refugee minors in Sweden [10].

In the intervention schools, youth who score 17 or above on the CRIES-8, which is the cut-off for PTSD symptoms, will be invited to participate in the TRT programme. The wait-list control schools will be informed about which youth scored above the cut-off of the CRIES, in order to provide services as usual. Furthermore, teachers in intervention schools will be offered the INSETT programme. The teachers in the wait-list control schools will be offered INSETT when all T3 follow-up data has been collected.

**Fig. 2:**
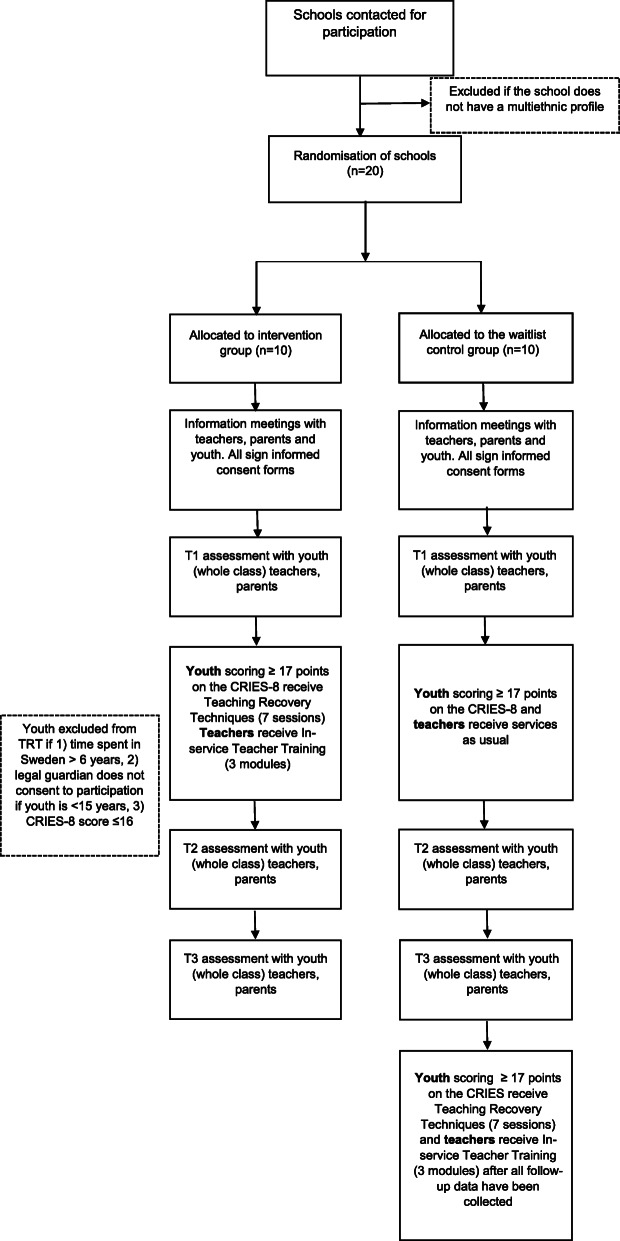
Flowchart of trial procedures

A Flow-chart for the trial procedure is shown in Fig. 2.

### Intervention

The intervention of this study comprises two programmes, TRT and INSETT (described below), which are delivered to the intervention group as well as to the wait-list control group, at a later stage.

### Teaching Recovery Techniques

Teaching Recovery Techniques (TRT) is a manualised group intervention, developed by the Children and War Foundation [23]. It is targeted to children and adolescents with PTSD symptoms and is based on TF-CBT, which is agreed to be the method of choice for reducing PTSD symptoms in these populations [21]. Two group leaders who have completed a three-day training programme, provided by the Swedish non-government organization Children’s Right in Society, deliver TRT over seven consecutive weeks. TRT involves two caregiver sessions and seven youth sessions, where each session is two hours long. Caregiver sessions include an introduction to TRT, an overview of the content in the youth sessions, psychoeducation, and information on how to support the youth and seek care if needed. Youth sessions focus on psychoeducation, intrusion, arousal, avoidance, and coping strategies. Each session involves skills training, rehearsal of skills, and homework. The first youth session focuses on group members getting to know one another whereas the final session acts as a closing in which participants consolidate learning and talk about their experience of taking part in the programme.

### In-service Teacher Training

In-service Teacher Training (INSETT) was developed by the Norwegian Centre for Violence and Traumatic Stress Studies (NKVTS) in Norway, and the Augeo Foundation in the Netherlands [32, 33]. The programme is designed to provide teachers with information and training related to trauma and the refugee experience to improve their cultural competency, sense of professional self-efficacy, and interrelationships with parents. It runs over a period of 10–12 weeks and consists of three interrelated course modules. An essential part of the programme is an online course module to be completed individually, including eight sections totalling four to five hours of study. Topics include trauma and stress, the therapeutic window of tolerance, self-regulation and coping, and identity and belonging. Each section provides theory, case histories, exercises, and recommendations for further reading [33].

The remaining two modules consist of whole-day seminars delivered in a group setting. One seminar takes place prior to the online course and introduces the course structure along with fundamental terms and elementary information about the refugee experience. The other seminar takes place after the online course has been completed. It allows participants to consolidate learning and to share their experiences of the intervention.

### Outcome measures

Multiple measures will be used to collect data from youths, parents/guardians, and teachers at three time points: T1 (pre-intervention), T2 (post-intervention) and T3 (three months follow-up), see Fig. 1. Below, we outline the primary and secondary outcomes for each participant group.

### Primary outcomes

#### Adolescents who receive TRT

For adolescents who receive TRT, the primary outcome is PTSD symptoms measured using the Children’s Revised Impact of Event Scale (CRIES-13) [35]. The CRIES-13 includes 13 self-report items on intrusion, avoidance and arousal which are answered on a scale from 0 (not at all) to 1, 3 and 5 (often). Total scores on the scale range from 0 to 65 with a cutoff score of 30 or above. The scale has been shown to have good internal consistency, and to successfully categorize more than 75% of children with and without a PTSD diagnosis [35, 37].

#### Parents/guardians whose children receive TRT

For parents/guardians whose children receive TRT and who attend TRT as caregivers, the primary outcome is mental health, as measured using the General Health Questionnaire (GHQ-12) [38] which screens for non-specific mental health problems using 12 self-report items on a 4-point scale from 0 to 3. This measure has shown good psychometric properties among adults in a large German sample [39] and has specifically shown excellent discriminant validity in a Swedish context [40].

#### Teachers who receive INSETT

For teachers who receive INSETT, the primary outcomes are parent-teacher collaboration and multicultural competence. Parent-teacher collaboration is measured using the Trust Scale (TS) [41, 42]. TS measures levels of trust between parents and teachers, including the elements of predictability, dependability, and faith, by asking participants to report on their level of agreement with 13 statements about parents’ performance and attitudes from 0 *(strongly disagree)* to 3 *(strongly agree).* The scale has demonstrated psychometric properties elsewhere [43].

Multicultural competence is measured by the Teacher Multicultural Attitude Scale (TMAS) [44] which includes 20 items for teachers to self-report about the cultural competence of their teaching and attitudes about multiculturalism on a scale of 1 (*strongly disagree*) to 5 (*strongly agree*). The scale has demonstrated both validity and internal consistency in separate samples of teachers [44].

#### Adolescents whose teachers receive INSETT

For adolescents who do not receive TRT but whose teachers participate in INSETT, the primary outcome is school belonging, which will be measured using 9 items of the 18-item Psychological Sense of School Membership Scale (PSSMS) [45]. The PSSMS captures student perceptions about caring relationships, acceptance, and rejection within their school [46] and uses a 5-point response scale from 1 *(not at all true)* to 5 *(completely true)*. The measure has demonstrated both high internal consistency and criterion validity in previous research [47].

### Secondary outcomes

#### Adolescents who receive TRT

For adolescents who receive TRT, the secondary outcomes include additional aspects of mental health problems, namely internalizing and externalizing problems, symptoms of depression, anxiety symptoms, general well-being and life satisfaction. Internalizing and externalizing problems are measured using the Strengths and Difficulties Questionnaire (SDQ) [48]. The SDQ is a widely known measure which comprises 25 items that can be divided into five subscales: emotional symptoms, hyperactivity, conduct problems, peer problems, and prosocial behavior. Each item is rated on a scale from 0 to 2 and each subscale has a total score range of 0–10. A total difficulties score can also be calculated as the sum of the emotional, conduct, hyperactivity and peer problems subscales, ranging between 0 and 40. The SDQ has previously demonstrated adequate psychometric properties in research on adolescents [48–50].

Symptoms of depression will be measured using the Patient Health Questionnaire-9 (PHQ-9) [51]. Participants report how often they have experienced nine depression symptoms in the previous two weeks on a scale from 0 (*not at all*) to 3 (*nearly every day*). The PHQ-9 has been validated among adolescents and has been shown to have strong construct validity and appropriate sensitivity and specificity [52].

Furthermore, anxiety symptoms will be assessed through the Generalized Anxiety Disorder scale (GAD-7) [53], a 7-item self-report measure originally developed to screen for generalized anxiety disorder. Individual items are rated according to the frequency of their occurrence during the past 2 weeks on a scale from 0 (*Not at all*) to 3 (*Nearly every day*). The GAD-7 has shown adequate reliability and validity in previous studies [54].

General well-being and life satisfaction will be assessed using the Cantril ladder [55]. The youth will be presented a picture of a ladder and asked to think about their life right now and place themselves on the ladder, choosing a number from 0 to 10 where 0 indicates the worst possible life satisfaction and 10 indicates the best. The Cantril Ladder measure will be used by the group leaders during the TRT sessions as part of a safety protocol and a check-in for the youth’s wellbeing.

In addition to mental health problems, other secondary outcomes for youth who receive TRT include number of stressors in daily life, social support, and resilience, perceived discrimination, school belonging and executive functioning. The Daily Stressors Scale for Young Refugees (DSSYR) is a 7-item self-report questionnaire that measures to what extent material stressors (insufficient housing, medical care, clothing/food and money) were experienced by the participants during the previous month; the questionnaire uses a five-point Likert scale from 1 (*not*) to 5 (*very much*) [6]. According to our knowledge, the psychometric properties of DSSYR have not yet been assessed in previous research. The Multidimensional Scale of Perceived Social Support (MSPSS) [56, 57] will be used to assess social support. The MSPSS is a 12-item self-report questionnaire designed to measure perceived social support from three sources: family, and friends, using a five-point Likert scale from 0 (*strongly disagree*) to 5 (*strongly agree*). The scale has established reliability and strong factorial validity in previous research [56, 58]. Also, the existence of interethnic friendships and friendship satisfaction, will be assessed through specific items developed for this study. Resilience will be measured by the Child and Youth Resilience Measure (CYRM-12) [59]. The CYRM is a 12-item self-report measure exploring the resources, i.e., individual, relational, communal and cultural, available to youth, that may bolster their resilience. It uses a 5-point Likert-scale ranging from 1 (*not at all*) to 5 (*a lot*), where higher scores equal to higher degree of higher resilience. Research has shown good reliability and sufficient content validity of the CYRM-12, when used among adolescents [59].

Furthermore, the 9-item scale Discrimination at Work/School of the Perceived Ethnic Discrimination Questionnaire-Community Version (PEDQ-CV) [60] will be used as a measure of experience of perceived discrimination. This scale includes items that refer to experiences of discrimination from school/work staff, classmates/colleagues or from other parts of society, and that are rated on a 4-point scale ranging from 1 *(never happened)* to 4 *(always)*. Both the scale and the full PEDQ instrument have established good reliability and construct validity [60].

Also, 9 items of the 18-item Psychological Sense of School Membership Scale (PSSMS) [45], as described above, will be used as a measure of feelings of belonging at school.

Executive functioning will be measured using the Amsterdam Executive Function Inventory (AEFI) [61] on which youth report perceptions of their own executive functions. The AEFI is a brief self-report questionnaire to assess three components of the executive aspects of daily life behavior, namely Attention, Self-Control and Self-Monitoring, and Planning and Initiative. The scale includes 14 items, which are rated on a 3-point Likert scale where 1 = “not true,” 2 = “partly true,” and 3 = “true”. Adequate construct validity and reliability of the AEFI has been demonstrated [61].

#### Parents/guardians whose children receive TRT

For parents/guardians whose children receive TRT and who attend TRT as caregivers, secondary outcomes include PTSD symptoms, general health, teacher-parent collaboration, perceived discrimination and social support. Outcomes are measured using the Posttraumatic Stress Disorder-8 (PTSD-8) [62] which screens for PTSD symptoms using eight items aligned to the Diagnostic and Statistical Manual of Mental Disorders-IV (DSM-IV) criteria for PTSD. Its items are rated on a four-point Likert scale from 1 *(not at all)* to 4 *(all the time)*. The PTSD-8 Questionnaire has established adequate psychometric properties in various samples [62].

An additional one-item on general health from the Short Form Health Survey (SF-36) [63] is also used as an outcome measure. The SF-36 is a multi-purpose, short-form health survey, containing 36 questions. The item used in the current trial is rated on a scale from 1 to 5, where 1 equals excellent and 5 equals poor. Overall, the SF-36 has demonstrated adequate psychometric properties in previous research [64].

To measure parent-teacher collaboration, the TS scale is used, as described above, but parents report on their beliefs about teachers [41, 42]. Experiences of discrimination are measured using the PEDQ-CV [60], and parents’ experiences of social support are measured using the Enriched Social Support Instrument (ESSI) [65]. The ESSI is a 7-item self-report survey that assesses four attributes of social support: emotional, instrumental, informational, and appraisal. Items are rated on a scale from 1 (*none of the time*) to 5 (*all of the time*). Due to its brevity and psychometric properties, the ESSI has been deemed as an excellent screening tool for social support [66].

#### Teachers who receive INSETT

For teachers who receive INSETT, secondary outcomes include teachers’ self-efficacy, stress symptoms, work exhaustion/burnout, work engagement, and classroom atmosphere. Self-efficacy is measured using the Teachers’ Sense of Efficacy Scale (TSES) [67]. The TSES directs teachers to respond to 12 items measuring their self-efficacy, given their current ability, resources, and opportunities in their present position. Participants respond to items on a scale from 1 (*none at all*) to 9 (*a great deal*). The TSES has established good reliability and factor structure in previous research [68].

Stress symptoms are measured by the Single Item Stress Index (SISI) [69], containing the following item “Stress means a situation in which a person feels tense, restless, nervous or anxious or is unable to sleep at night because his/her mind is troubled all the time. Do you feel this kind of stress these days?” The response is rated on 5-point Likert scale from 1 (*not at all*) to 5 (*very much*). The SISI has shown satisfactory content, criterion, and construct validity among adults in various samples [69].

Furthermore, work exhaustion/burnout will be measured by the Bergen Burnout Inventory (BBI) [70]. The BBI comprises 15 self-report items that measure burnout through three scales: exhaustion at work, cynicism toward the meaning of work and the sense of inadequacy at work. All items are rated on a 6-point Likert-type scale ranging from 1 *(completely disagree)* to 6 *(strongly agree).* The instrument has demonstrated reliability and construct validity among adults in various samples [70]. In the current study, we will use the work exhaustion scale, comprising five items.

Also, work engagement will be measured by items of the Utrecht Work Engagement Scale (UWES) [71]. The UWES comprises 17 self-report items that can be divided into three subscales that reflect the underlying dimensions of engagement: Vigor, Dedication and Absorption. All items are scored on a 7-point frequency rating scale ranging from 0 (*never)* to 6 *(always).* The UWES has been found to have good internal consistency, factor structure and test-retest reliability [72]. In this study, the items of the Dedication scale will be used to assess work engagement.

Finally, teachers’ perceptions of classroom atmosphere are measured using a scale developed in a psychosocial school intervention study [73]. The scale comprises three items on bullying, disruptive behavior and enjoyment in the classroom. Items are rated on a scale from 1 *(a little)* to 5 *(a lot)*. According to our knowledge, the psychometric properties of this scale have not yet been assessed in previous research.

#### Adolescents whose teachers receive INSETT

Additional aspects of social support, perceived discrimination and school belonging are the secondary outcomes for youth who do not receive the TRT programme but whose teachers received the INSETT programme. Social support is measured by the MSPSS [56, 57], as described above. Experience of discrimination are measured by the PEDQ-CV [60]. Finally, the existence of interethnic friendships and friendship satisfaction is measured by questions developed for this study.

### Other measures

The study will also use items in order to gather demographic information. For example, items for youth and parents include information on age, gender, ethnicity, time spent in Sweden and asylum status. Additionally, items for teachers include information on age, gender, educational background, teaching experience and current teaching situation, e.g., subjects, grades and number of students. This data will be used for descriptive purposes to, examine the extent to which demographic characteristics are balanced between study groups, carry out attrition analyses and identify subgroups. The demographics questionnaire will be administered at T1.

Furthermore, during the TRT sessions, the Columbia-Suicide Severity Rating Scale (C-SSRS) Screen Version will be used part of a safety protocol for participants who indicate they have had thoughts they would be better off dead (ninth item on the PHQ-9) or ‘suffering’ on the Cantril Ladder (i.e. a score of 4 or below). The C-SSRS Screen Version is a 6-item structured interview or self-report measure that assesses the presence and severity of suicidal ideation and behavior and is commonly regarded as the “gold standard” for assessment of suicidal ideation and behavior in clinical trials [74]. The safety protocol will be used in order to signpost or refer the youth to another service when needed. Frequency of using the protocol will be reported and any spontaneously reported adverse events will be recorded and managed accordingly by clinical expertise of the research team. Additionally, resources needed to deliver the various parts of the intervention will be collected based on professionals’ time, costs for materials handed out to participants and participant’s time.

No biological specimens will be collected as part of the trial.

### Sample size

Power calculations were conducted. With alpha set at 0.05, power at 0.80, and Rho = 0.05 (the intra-cluster correlation), a sample size of at least 40 clusters (i.e., schools) with 25 youths each was identified as necessary. Therefore, 500 youths per study arm (i.e., intervention or wait-list control) are needed, resulting in 1000 total participants. In order to account for an expected dropout rate of 20%, 1250 youths will be recruited in total.

In order to facilitate the recruitment of such a large number of participants, recruitment will be split between our Swedish study team and our research partners in Finland. The Swedish site will recruit 10 intervention clusters 10 waitlist-control clusters and with 35 youths in each cluster, resulting in 700 individuals total. Work planned and conducted by the Finnish partner site is outside the scope of this study protocol and will be reported on independently by their team.

### Randomization

A computed-generated randomization sequence will be used in order to allocate schools into the intervention and wait-list control arms, respectively. Block randomization will be used to ensure equivalence between intervention and waitlist-control schools. Randomization will be conducted prior to T1 and through an online, third party central randomization service named sealed envelope (www.sealedenvelope.com). One person of the research team, blinded to the schools, will run the randomization process. Researchers and schools will not be blinded to the group allocation, however. All schools will be informed on which group they have been allocated to.

### Data collection

Outcome data will be collected at three time points using a secure online platform [75]. Paper surveys will also be made available to participants who may lack access to technology. Surveys are available in 26 languages. To promote and monitor adherence to the core design of the intervention, a fidelity checklist on paper will be distributed to group leaders, who will return the list to the research team after the program has been finished.

All participants will be given an anonymous identity number. This study is part of the larger EU Horizon 2020 project and outcome data will be shared with partner institutions including the Ghent University (Belgium), KU Leuven (Belgium), Copenhagen University (Denmark), University of Tampere (Finland), Norwegian Centre for Violence and Traumatic Stress Studies (NKVTS; Norway), Sussex University (UK), who are investigating other intervention programmes; discussion of these institutions’ work is outside the scope of this study protocol. Data will be exported/inputted into SPSS files for analyses, and saved on the university server, which is automatically backed up. All data will be stored and managed according current regulation on personal data management.

Qualitative focus group discussions with teachers, stakeholders and TRT group leaders will be carried out to explore how the intervention has been implemented, and which factors that facilitated or hindered the implementation process. All the focus groups will be transcribed verbatim and analyzed separately using content analysis [76]. The analysis will start by reading all transcribed data inductively several times. Then, data will be read word by word to derive codes by highlighting words from the text that appeared to capture key thoughts or concepts. These text segments will be further organized and grouped to constitute categories.

All participants will be informed that the data provided will be treated confidentially and will be made aware that in published reports the results will be reported anonymously and at a group level, meaning that it will not be possible to identify any individual or attribute any information to them. Participants will be informed that if they disclose anything concerning their personal safety, the safety protocol will be implemented.

### Statistical analyses

Baseline and demographic characteristics will be summarized using means and standard deviations (or medians or and interquartile ranges in case of skewed distributions) for continuous variables and frequencies and percentages for categorical variables. Various strategies will be employed in order to minimize the amount of missing data (e.g. sending out reminders for completing follow-ups) and reasons for drop-out will be reported. The possible impact of missing data will be examined via sensitivity analyses of augmented data sets and missing data will be handled through modern imputation techniques such as multiple imputation.

Outcomes for the trial arms will be compared using linear mixed models or ANCOVA repeated measures. The comparison of the trial arms will use the intention-to-treat framework where all participants are analyzed according to the trial arm they were randomized to, regardless of whether they received the intervention. Additionally, we will utilize the Complier Average Causal Effect (CACE) or per protocol frameworks where participants are analyzed according to which intervention they received [77].

For mental health outcome measures, we will also use the Reliable Change Index (RCI) and Clinically Significant Change (CSC) approaches, classifying participants as ‘recovered’, ‘improved’, ‘unchanged’ or ‘deteriorated’ [78]. These approaches comprise measures of whether the change in scores is larger than expected due to outcome measure reliability and whether participants shifts from a clinical state to a non-clinical state. The proportions of classified participants will be compared across the trial arms.

Furthermore, fidelity, in terms of adherence and dose of the intervention, will be summarized using descriptive statistics. We will also compute moderation analyses in order to examine the associations between improvement status and participants’ characteristics (e.g., age and gender). Effect size measures such as Cohen’s d will be calculated in order to describe intervention effects.

For the economic evaluation, the outcomes and costs between the intervention and control groups will be compared using bootstrapped mixed model analyses. Two types of evaluations will be performed: (i) a cost-utility analysis with outcomes measured in Quality Adjusted Life Years (QALYs); and (ii) a cost-effectiveness analysis with proportion of participants classed as treatment success expressed as incremental cost-effectiveness ratios [79]. SDQ score will be translated to utility values [80]. Health gains in term of QALYs will be estimated using any change in utilities, at baseline and respective assessment points.

The cost-effectiveness ratios describe: (i) the price for one additional life year with full health gained, and (ii) the price to get an additional successfully treated participant. All statistical analyses will be computed using the SPSS and R softwares.

## Discussion

This study will address knowledge gaps in the scientific community by evaluating the school-based intervention comprising the TRT and INSETT programmes in an applied, school-based setting, using a long-term follow-up (i.e. three months) which allows for the investigation of long-term intervention effects. Data will be collected using multiple measures from multiple sources (i.e., parents/guardians, teachers and youth) consistent with best practices [81] and most of the measures used have adequate psychometric properties. The cluster randomized design has less statistical power than an ordinary randomized trial. However, it was deemed necessary to randomize schools in order to ensure that all students within the school would have the same opportunities for participation and not feel alienated or traumatized by not receiving access to the intervention at the same time as other classmates. Randomizing schools also reduces contamination and network effects within schools in comparison to randomization of individuals. Another potential limitation might be difficulties in receiving informed consent from parents/guardians. Therefore, the research team will work closely with each class mentor to collect consent from parents/guardians. The results of this study will be used to inform future decisions related to the provision of mental health and wellbeing interventions to refugee youth.

### Trial status

Protocol version 1, 2020-07-06. Recruitment of participants began 13/08/2018 and will continue to 31/12/2020.

## Supplementary Information

**Additional file 1.**

## Data Availability

The results from this trial are due to be submitted for publication during spring 2021. Authorship will be granted for substantive contributions to the design, conduct, interpretation and reporting of the trial. The ultimate decision on authorship will be made by the Principal. Investigators (AS, ID). Publications will be made open access. The data sets generated during the current study will not be made open access, but can be made available upon request to the Principal Investigators (AS, ID) and according to the ethical approval.

## Abbreviations

AEFI: Amsterdam Executive Function Inventory
ANCOVA: Analysis of Covariance
BBI: Bergen Burnout Inventory
CACE: Complier Average Causal Effect
CHAP: Child Health and Parenting
CRIES: Children’s Revised Impact of Event Scale
CSC: Clinically Significant Change
C-SSRS: Columbia-Suicide Severity Rating Scale
CYRM: Child and Youth Resilience Measure
DSM-IV: Diagnostic and Statistical Manual of Mental Disorders-IV
DSSYR: Daily Stressors Scale for Young Refugees
ESSI: ENRICHED Social Support Instrument
GHQ-12: General Health Questionnaire
INSETT: In-service Teacher Training
MSPSS: Multidimensional Scale of Perceived Social Support
NKVTS: Norwegian Centre for Violence and Traumatic Stress Studies
PHQ-9: Patient Health Questionnaire-9
PEDQ-CV: Perceived Ethnic Discrimination Questionnaire-Community Version
PSSMS: Psychological Sense of School Membership scale
PTSD: Post-traumatic stress disorder
QALY: QualityAd justed Life Year
RCI: Reliable Change Index
RCT: Randomized Controlled Trial
RWS: RefugeesWellSchool
SDQ: Strengths and Difficulties Questionnaire
SISI: Single Item Stress Index
SPSS: Statistical Package for the Social Sciences
TF-CBT: Trauma-Focused Cognitive Behavioral Therapy
TMAS: Teacher Multicultural Attitude Scale
TRT: Teaching Recovery Techniques
TS: Trust Scale
TSES: Teachers’ Sense of Efficacy Scale
UWES: Utrecht Work Engagement Scale
