# Correction to: Evaluation of a school-based intervention to promote mental health of refugee youth in Sweden (The RefugeesWellSchool Trial): study protocol for a cluster randomized controlled trial

**DOI:** 10.1186/s13063-021-05840-2

**Published:** 2021-11-29

**Authors:** Natalie Durbeej, Serena McDiarmid, Anna Sarkadi, Inna Feldman, Raija-Leena Punamäki, Reeta Kankaanpää, Arnfinn Andersen, Per Kristian Hilden, An Verelst, Ilse Derluyn, Fatumo Osman

**Affiliations:** 1 Child Health and Parenting (CHAP), Department of Public Health and Caring Sciences, Uppsala University, BMC, Husargatan 3, 753 27 Uppsala, Sweden; 2 Faculty of Social Sciences, Psychology, FI- 30014, University of Tampere, Tampere, Finland; 3 Norwegian Centre for Violence and Traumatic Stress Studies, NO-0409 Oslo, Norway; 4 Faculty of Psychology and Educational Sciences, Department of Social Work and Social Pedagogy, Centre for the Social Study of Migration and Refugees, Ghent University, Ghent, Belgium; 5 School of Education, Health and Social Studies, Dalarna University, 791 88 Falun, Sweden

**Correction to: Trials 22, 98 (2021).**

**https://doi.org/10.1186/s13063-020-04995-8**

Following the publication of the original article [1], we were notified of the below corrections:

**1) Page 2.**

The following paragraph:

An intervention targeting teachers working with refugee youth is the In-service Teacher Training (INSETT) programme [32, 33], aiming to enhance teachers’ insights into how refugee experiences may affect young people’s psychosocial well-being and school functioning upon resettlement in a new country [29, 32]. It allows teachers to better understand and support young refugees at school through encouraging positive interethnic relationships and strengthening school belonging, as well as fostering supportive interrelationships with parents, caregivers or guardians to promote school involvement. In other words, INSETT seeks to make teachers and schools (more) ‘refugee competent’ [20]. No previous research has evaluated the INSETT programme in a school setting.

Should read:

An intervention targeting teachers working with refugee youth is the In-service Teacher Training (INSETT) programme [32, 33], aiming to strengthen teachers’ competence on how refugee experiences may affect school functioning and psychosocial wellbeing in youth upon resettlement in the new host country. [29, 32]. The programme enhances teachers’ ability to support young refugee students through promoting interethnic relationships with peers, school belonging, and supportive relationships between teachers and parents/caregivers [20]. No previous research has evaluated the INSETT programme in a school setting.

**2) Page 5.**

The following sentence:

In-service Teacher Training (INSETT) was developed by the Norwegian Centre for Violence and Traumatic Stress Studies (NKVTS) in Norway, and the Augeo Foundation in the Netherlands [32, 33].

Should read:

In-service Teacher Training (INSETT) was developed by Lutine de Wal Pastoor at the Norwegian Centre for Violence and Traumatic Stress Studies (NKVTS) in Norway, and the Augeo Foundation in the Netherlands [32, 33].

**3) Page 5.**

The following paragraph:

It runs over a period of 10–12 weeks and consists of three interrelated course modules. An essential part of the programme is an online course module to be completed individually, including eight sections totalling four to five hours of study. Topics include trauma and stress, the therapeutic window of tolerance, self-regulation and coping, and identity and belonging. Each section provides theory, case histories, exercises, and recommendations for further reading [33].

Should read:

It comprises three course modules and runs over a 10–12 week period. An essential part of the programme is an online course module to be completed individually, including eight sections totalling four to five hours of study. The online module topics includes traumatic experiences, stress, self-regulation, coping strategies, identity and belonging. Each section provides theory, various exercises and case studies, as well as references for further reading [33].

**4) Page 12, “**Working paper 20.02.2019” was added to Reference no 32.

The original article has been corrected.
